# Exercise-induced responses in matrix metalloproteinases and osteopontin are not moderated by exercise format in males with overweight or obesity

**DOI:** 10.1007/s00421-023-05133-3

**Published:** 2023-01-17

**Authors:** Aaron Raman, Jeremiah J. Peiffer, Gerard F. Hoyne, Nathan G. Lawler, Andrew Currie, Timothy J. Fairchild

**Affiliations:** 1 Discipline of Exercise Science, Murdoch University, Murdoch, Australia; 2 Centre for Healthy Ageing, Health Futures Institute, Murdoch University, Murdoch, Australia; 3 School of Health Sciences and Physiotherapy, Notre Dame University, Fremantle, Australia; 4 Australian National Phenome Centre, Health Futures Institute, Murdoch University, Murdoch, Australia; 5 The Centre for Molecular Medicine and Innovative Therapeutics, Health Futures Institute, Murdoch University, 90 South Street, Murdoch, WA 6150 Australia

**Keywords:** Obesity, Cardiovascular disease, Glucose regulation, Interval training, High intensity

## Abstract

**Purpose:**

Matrix metalloproteinase-2 (MMP-2) and -3 (MMP-3), and osteopontin (OPN) are associated with adipose-tissue expansion and development of metabolic disease. The purpose of the current study was to assess the circulating concentration of these markers, along with adiponectin and glucose concentrations, in response to acute exercise in individuals with overweight or obesity.

**Methods:**

Fourteen sedentary males with overweight or obesity (29.0 ± 3.1 kg/m^2^) completed two separate, 3-day trials in randomised and counterbalanced order. An oral glucose tolerance test (OGTT) was performed on each day of the trial. Day two of each trial consisted of a single 30 min workload-matched bout of either high-intensity interval exercise (HIIE; alternating 100% and 50% of peak pulmonary oxygen uptake, $\mathop {\text{V}}\limits^{.}$O_2peak_) or continuous moderate intensity (CME; 60% $\mathop {\text{V}}\limits^{.}$O_2peak_) cycling completed 1 h prior to the OGTT. Glucose and physical activity were continuously monitored, while MMP-2, MMP-3, OPN and adiponectin were measured pre-, 0 h post-, 1 h post- and 25 h post-exercise.

**Results:**

Exercise transiently increased MMP-3 and decreased OPN (both *p* < 0.01), but not MMP-2 or adiponectin. There were no differences in the response of inflammatory markers to the different exercise formats. Exercise increased mean daily glucose concentration and area under the glucose curve during the OGTT on Day 2 and Day 3 (main effect of time; *p* < 0.05).

**Conclusion:**

Acute cycling exercise decreased OPN, which is consistent with longer term improvements in cardiometabolic health and increased MMP-3, which is consistent with its role in tissue remodelling. Interestingly, exercise performed prior to the morning OGTT augmented the glucose concentrations in males.

**Trial registration:**

ACTRN12613001086752.

## Introduction

Obesity and its determinants have now been identified as risk factors for three of the four leading causes of non-communicable diseases worldwide (Swinburn et al. 2019). With an estimated two billion people worldwide living with obesity in 2015 (Swinburn et al. 2019), there is a need to better understand the early developmental stages of obesity and obesity-related conditions. Obesity is characterised by adipose tissue expansion with infiltration of macrophages expressing pro-inflammatory cytokines (Nomiyama et al. 2007; Asghar and Sheikh 2017; Wensveen et al. 2015). These pro-inflammatory cytokines can impair insulin signalling (Lazar 2005) leading to chronically elevated blood glucose concentrations, and contribute to microvascular and macrovascular complications in diabetes mellitus (Bagi et al. 2014; Knight and Imig 2007; Van Gaal et al. 2006; Wellen and Hotamisligil 2003). Microvascular and macrovascular complications largely account for the high rates of all-cause mortality in individuals with diabetes mellitus (Fox et al. 2007; Meshkani and Vakili 2016).

The events initiating the shift to the predominantly pro-inflammatory state in obesity remain unclear (Pirola and Ferraz 2017; Wang et al. 2017), although several candidates have emerged as potential contributors. A class of enzymes known as matrix metalloproteinases (MMPs) play important roles in the early stages of obesity-related disease progression (Bouloumie et al. 2001; Visse and Nagase 2003). Specifically, MMP-2 is released from adipose tissue during adipocyte differentiation, and is understood to regulate the vascular extracellular matrix remodelling during adipose tissue formation (Visse and Nagase 2003; Bouloumie et al. 2001). MMP-3, on the other hand, counteracts the role of MMP-2 by inhibiting adipocyte differentiation and halting hyperplastic adipose tissue expansion (Wu et al. 2017).

Osteopontin (OPN), a multifunctional protein shown to promote macrophage migration (Lund et al. 2013) is another potential candidate. OPN was shown to increase levels of the classically activated macrophages in adipose tissue, which are the primary source of circulating pro-inflammatory cytokines (Kahles et al. 2014; Nomiyama et al. 2007). OPN is a substrate for MMP-2 and MMP-3 (Lindsey et al. 2015), with resultant OPN peptides maintaining distinct biological functions.

Concentrations of serum MMP-2 (Derosa et al. 2008) and OPN are increased in patients with obesity and type 2 diabetes mellitus (T2D) (Daniele et al. 2014), while OPN has been implicated in mediating the relationship between inflammation, obesity and diabetes (Kahles et al. 2014). In contrast, the concentrations of MMP-3 (Goncalves et al. 2015) and the adipose tissue derived anti-inflammatory cytokine adiponectin (APN), which can increase the expression of MMP-3 (Tong et al. 2011), are reduced in patients with T2D. The increased concentrations of MMP-2 and reduced concentrations of MMP-3 in T2D are likely associated with hyperglycaemia (Death et al. 2003).

Exercise training plays an important role in decreasing visceral adipose tissue (Verheggen et al. 2016), and improving both acute and chronic glycaemia (Umpierre et al. 2011; Thomas et al. 2006). The response of MMPs to exercise have only been assessed in a few studies with humans, while the extent to which circulating MMPs respond to direct modifications in exercise variables (e.g., intensity, frequency, duration, type) remain largely unexplored in humans (Jaoude and Koh 2016). Further, the response of MMPs to exercise, remains equivocal in humans (Jaoude and Koh 2016). The inconsistent findings may be due to differences in exercise variables (e.g., resistance exercise versus aerobic exercise) and study-populations (non-obese, obese or T2D) (Jaoude and Koh 2016). Given the purported benefits of high-intensity intermittent exercise (HIIE) in regulating glucose concentrations and adiposity (Savikj and Zierath 2020), this study sought to determine i) the acute responses in MMP-2, MMP-3, OPN and APN; ii) the response in 48-h glucose profile, to a HIIE bout compared with an energy-matched moderate-intensity exercise bout. Due to the purported role of MMPs in adipose tissue remodelling and the importance of exercise in decreasing adipose tissue, this study recruited individuals with overweight or obesity. We hypothesised that exercise would reduce 48 h glucose concentrations, acutely increase OPN and MMP-3, and acutely decrease MMP-2, and this would be more pronounced following HIIE.

## Methods

### Participants

Fourteen sedentary (≤ 2 bouts of low-to-moderate intensity exercise per week) and overweight/obese (BMI > 25 kg/m^2^) males were recruited from the local community via advertisements posted on public noticeboards. The sample size (*n* = 14) estimate was calculated using a medium effect size (*f* = 0. 25) with 80% power and an α error probability of 0.05 using g*power (correlation among repeated [4] measures was set to 0.75). Participants were deemed eligible if they were aged between 18 and 44 years, had not been previously diagnosed with T2D (fasting plasma glucose < 7 mmol/L) and had not smoked within the previous 6 months. Participants were excluded if they were taking anti-inflammatory medication, glucose controlling drugs (i.e. metformin), had uncontrolled hypertension, and had either an acute or chronic inflammatory disease/infection. Participants were pre-screened for exercise using the Exercise Science and Sport Science Australia medical screening tool.

### Study design

The study adopted a randomized cross-over design as previously reported (Raman et al. 2018). All participants attended the Exercise Physiology labs at Murdoch University, Perth, Western Australia, for a total of seven visits (introductory session; two experimental phases comprising three visits each). The introductory session (visit 1) involved the consent process, a familiarization session and assessment of fitness (peak oxygen consumption, $\mathop {\text{V}}\limits^{.}$O_2peak_; maximal aerobic power assessment). The two experimental phases comprised three study days each, with each phase completed in randomized order. The randomization sequence was generated using a computer-generated random number list comprising 1s and 2s. The allocation of participants was conducted by a researcher not involved in the delivery of the intervention (TJF), by placing pre-numbered cards into opaque, sealed envelopes and recording the study ID’s. Participants were informed of the possible risks associated with the study before giving written informed consent. Ethics approval was granted by Human Research Ethics Committee at Murdoch University and the trial registered with Australian New Zealand Clinical Trials Registry (ACTRN12615000613505).

### Introductory session

On arrival, participants were fully informed of all study procedures and written informed consent obtained. Body height, body mass and waist circumference measures were taken, and participants then shown the finger-stick procedure for collecting capillary blood for determination of blood glucose using a glucometer (Accu-Chek Go, Roche, Mannheim, Germany) required for the calibration of the continuous glucose monitor. Body composition (body fat percentage and visceral fat volume) was measured using the Dual-Energy X-ray Absorptiometry (DXA; Hologic Discovery QDR series, Hologic Inc., Bedford, MA, USA).

Thereafter, participants completed an incremental cycling test (50 W; with 25 W increments every 2 min) until volitional exhaustion on an electronically braked Velotron cycle ergometer (RacerMate, Seattle, WA, USA) for measures of $\mathop {\text{V}}\limits^{.}$O_2peak_. During the incremental cycling test, expired ventilation was collected as 15 s mean values using the Parvomedics TrueOne metabolic cart (Parvomedics, Sandy, UT, USA) and heart rate (Polar T31, Kempele, Finland) was collected at 1 Hz. Peak $\mathop {\text{V}}\limits^{.}$O_2_ was determined from the highest 30 s average $\mathop {\text{V}}\limits^{.}$O_2_ during the final stages of the incremental test. Heart rate and Borg’s (Borg 1970) rating of perceived exertion (RPE) was collected throughout the maximal graded exercise test. This session was completed at least 3 days prior to the first experimental phase.

### Experimental trials

On day one of the experimental phase, participants reported to the laboratory (~ 0700 h) for the insertion of the continuous glucose monitoring system (CGMS; iPro2, Medtronic, Northridge, CA, USA). The sensor was inserted into an area of subcutaneous fat in the abdomen as recommended by the manufacturer. Participants were provided with a glucose meter (Accuchek Go, USA) to collect four finger-prick blood samples per day to calibrate the glucose monitor and a triaxial accelerometer (ActiGraph GT3X, Actigraph L.L.C, FL, USA) to assess physical activity patterns over the 3-day period. Participants then conducted an oral glucose tolerance test (OGTT; 0740–0930 h) which included consuming a 75 g glucose drink, followed by two hours of quiet resting. Participants were then provided with a standardized breakfast (Total kJ content is 2295.6 kJ; Up and Go, Sanitarium, Australia; two muesli bars, Kellogg’s, Australia) before leaving the laboratory, as previously described (Raman et al. 2018). Food intake for the remaining portion of the day was recorded in a food diary and repeated during the second phase (alternate exercise protocol).

On day two, participants arrived fasted at the laboratory at 0600 h (overnight fast; water permitted). A venous blood sample was collected into vacutainer tubes (EDTA, ethylenediamine tetraacetic acid; SST, Spray-coated with silica; Becton Dickinson, NJ, USA) from the antecubital vein of the forearm. Participants then completed a 5-min warm up at 75 W before completing the assigned exercise condition. Immediately upon the cessation of exercise, a second venous blood sample was collected, followed by a recovery where participants were advised to relax and remain seated prior to the third venous blood sample being collected one hour following cessation of exercise. Participants then repeated (day 2) the 75 g-OGTT. Thereafter, participants were provided with a standardized breakfast (total energy content: 2295.6 kJ; Up and Go, Sanitarium, Australia; two muesli bars, Kellogg’s, Australia) prior to leaving the laboratory.

On the subsequent day, participants reported to the laboratory in a fasted state and had their final (25 h) venous blood sample collected, which was performed at the same time as the previous days. Participants then completed their final (day 3) 75 g-OGTT.

### Exercise conditions

Each of the two experimental phases followed an identical three-day design, with the exception being the type of exercise performed on day two. Day two comprised either a continuous moderate intensity exercise bout (CME; 30 min at 60% of $\mathop {\text{V}}\limits^{.}$O_2peak_), or a high intensity intermittent exercise (HIIE) bouts. The HIIE bout comprised six efforts at high- (1 min at 100% $\mathop {\text{V}}\limits^{.}$O_2peak_) and low- (4 min at 50% $\mathop {\text{V}}\limits^{.}$O_2peak_) intensity which lasted 30 min in duration. Participants were advised to maintain a cadence between 80 and 110 revolutions per minute (rpm) during the intense bouts and to maintain a comfortable cadence during the recovery period. This exercise protocol was based on a previously published protocol from our group (Sim et al. 2014). Heart rate and RPE were recorded throughout each exercise trial.

### Blood sampling

Serum samples (SST; 5 ml) were left for at least 30 min to clot at room temperature, before being centrifuged at 1300×*g* for 15 min. Plasma samples (EDTA; 6 ml) were spun immediately after collection at 1300 g for 15 min. The supernatant was then aliquoted in triplicate and stored at − 80 °C for later analysis. Samples were batch analysed for plasma glucose using a commercial kit (GAHK20, Sigma-Aldrich, MO, USA); serum APN using a multiplex cytokine assay (Bio-Plex Pro Human Diabetes Assay); MMP2, MMP-3 and OPN (Isoform B) (Bio-Plex, 37-plex Human Inflammation Panel) using the MAGPIX multiplex reader (Bio-Rad, Richmond, CA, USA).

### Physical activity monitoring

A triaxial accelerometer (ActiGraph GT3X Activity Monitor; ActiGraph LLC, FL, USA) was provided to participants to record daily physical activity. The Actigraph was attached to an adjustable elastic band and worn on the right hip for three consecutive days, except during water-related activities (e.g., swimming or showering). Physical activity data were then assessed using the ActiLife software (version 5.6.1; ActiGraph LLC, FL, USA). Data were recorded in 60 s epochs and to be included in the analysis, participants were required to wear the accelerometer for at least 10 h of valid wear time per day (Migueles et al. 2017). Freedson Adult (1998) cut points within the Actilife software were applied for sedentary (0–99 counts per minute) activity. Outcomes of interest included sedentary time (min/day), vector magnitude (counts per minute, CPM) and energy expenditure (kCal) which was estimated by the algorithm incorporated within the software, specifically set for adults.

### Data analyses

Data are presented as mean ± SD, unless otherwise stated. Raw data from the CGMS and Actigraph were inspected for missing values and outliers prior to time synchronization and analysis. The raw values from the CGMS were evaluated as described below. Differences in blood analytes (MMP-2, MMP-3, OPN, APN), AUC-glucose, heart rate, RPE, mean-daily physical activity levels were analysed using a linear mixed model (LMM) with repeated measures (time), with one between factor (trial: CME vs HIIE) and one within factor (time: pre-exercise, post-exercise, 1 h post-exercise and 25 h post-exercise) modelled as fixed effects (intercept modelled as random effects). The primary outcome of interest was the time by condition interaction, however, due to the time-points adopted, significant main effects of time were explored.

To avoid the problem of multiple point-wise comparisons over time, a bootstrapping analysis was used to assess the glucose values obtained from the CGMS. To this end, the blood glucose concentration at each time point on the comparator day (i.e., day 1 of each phase), was subtracted from the blood glucose concentration at each time point on the study day (i.e., Day 2 or Day 3 of each phase). This provided a difference or change score for each participant across the 2 days and enabled the calculation of a group-mean and 95% confidence interval (CI) bands over the course of the day. The change in glucose concentration was plotted against time (as mean ± 95% CI), wherein a significant response was defined as occurring when the lower 95% confidence limit for the curve was greater than zero, and a significant reduction in blood glucose was defined as occurring when the upper 95% confidence limit was less than zero (Kanaley et al. 2014). All statistical analyses were completed using commercially available software (SPSS 24 Windows; SPSS, Chicago, IL, USA). Significance was accepted at *p* ≤ 0.05.

## Results

The participants in the study were 27.4 ± 6.3 (mean ± SD) years old; classified as either overweight (*n* = 12) or obese (*n* = 2) by BMI (29.0 ± 3.2 kg/m^2^); with total body fat percent of 27.2 ± 4.2% and visceral fat of 613.2 ± 122.7 cm^3^. Participants were normoglycemic (fasting glucose: 5.2 ± 0.6 mmol/L) and had a VO_2_ peak of 35.7 ± 5.1 ml/kg/min. All participants completed the two experimental phases. One participant completed only five sprints during the HIIE bout, whilst one other participant completed all six sprints in the HIIE bout but did not complete the final 4 min (low-intensity portion of the protocol) of the bout. HIIE elicited a significantly higher mean heart rate and RPE (151 ± 33 bpm and 16 ± 2, respectively) than CME (137 ± 25 bpm and 14 ± 2, respectively).

All subjects provided valid wear time data on each of the days during the experimental phases (Table 1). Energy expenditure (kCal), total time in sedentary activity (Sed Time) and the vector magnitude (VM) were no different between days (*p* > 0.178 for all variables) or conditions (*p* > 0.196 for all variables).

**Table 1:** Continuous physical activity monitoring data stratified by condition (CME; HIIE) and measurement outcome

	CME			HIIE		
	kCal	Sed. time (min)	VM (cpm)	kCal	Sed. time (mins)	VM (cpm)
D1	401 (181)	881 (83)	7566 (1688)	504 (370)	813 (146)	9162 (5602)
D2	498 (312)	881 (192)	8697 (3402)	493 (485)	875 (179)	9037 (6683)
D3	546 (450)	840 (174)	9393 (6247)	629 (533)	821 (169)	10,137 (6646)

Energy expenditure expressed as kilocalories (kCal); Time (min) spent in sedentary activity: Sed. Time (min); Total vector magnitude (VM) expressed as counts per minute (cpm). Data are presented as mean (SD)

There were no significant differences. D1: day one; D2: day two; D3: day three

### Inflammatory cytokines

**Fig. 1:**
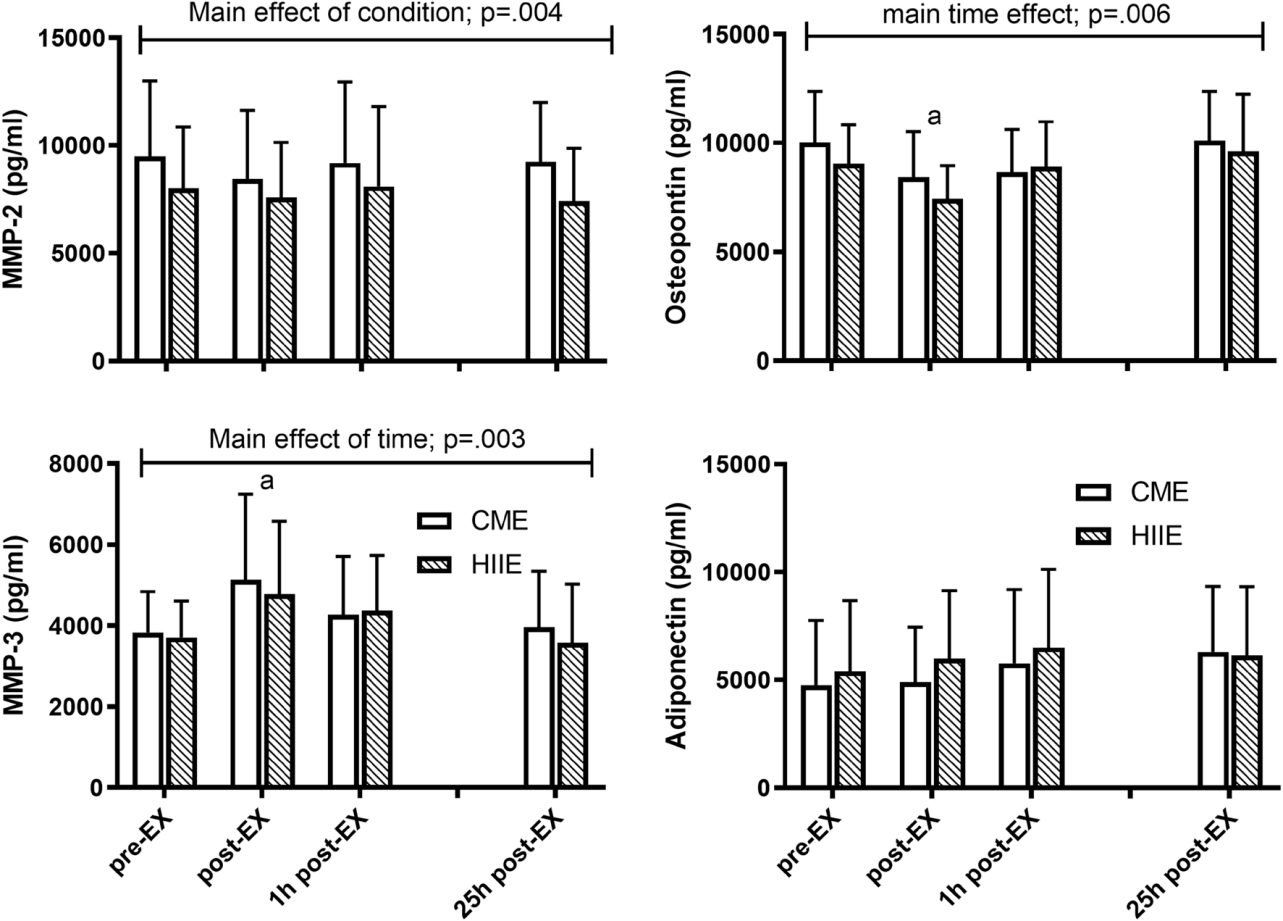
Adiponectin, matrix metalloproteinases (MMP-2, MMP-3) and osteopontin concentrations in response to exercise. Mean (± SD) concentration of MMP-2, MMP-3, osteopontin and adiponectin are presented pre-exercise (pre-EX), immediately post-exercise (post-EX), 1 h post-exercise (1 h post-EX) and 25 h post-exercise (25 h post-EX). MMP-3; a, significantly different than Pre (*p* = 0.001) and 25 h (*p* = 0.001). Osteopontin; a, significantly different than Pre (*p* = 0.006) and 25 h (*p* = 0.001)

Complete data from only ten participants were available for analysis of the MMP-2, MMP-3, APN and OPN (Fig. 1). There were no time-by-condition interactions observed for the markers of interest (MMP-2: *p* = 0.876; MMP-3: *p* = 0.886; OPN: *p* = 0.663; APN: *p* = 0.778). A main effect of time for MMP-3 (*p* = 0.003) and OPN (*p* = 0.006) were observed (Fig. 1). Exercise resulted in an acute increase in MMP-3, which was ablated by 1 h post-exercise. In contrast, OPN decreased significantly in response to exercise, returning to pre-exercise levels by 25 h post-exercise (Fig. 1).

### Glucose: continuous glucose monitoring

**Fig. 2:**
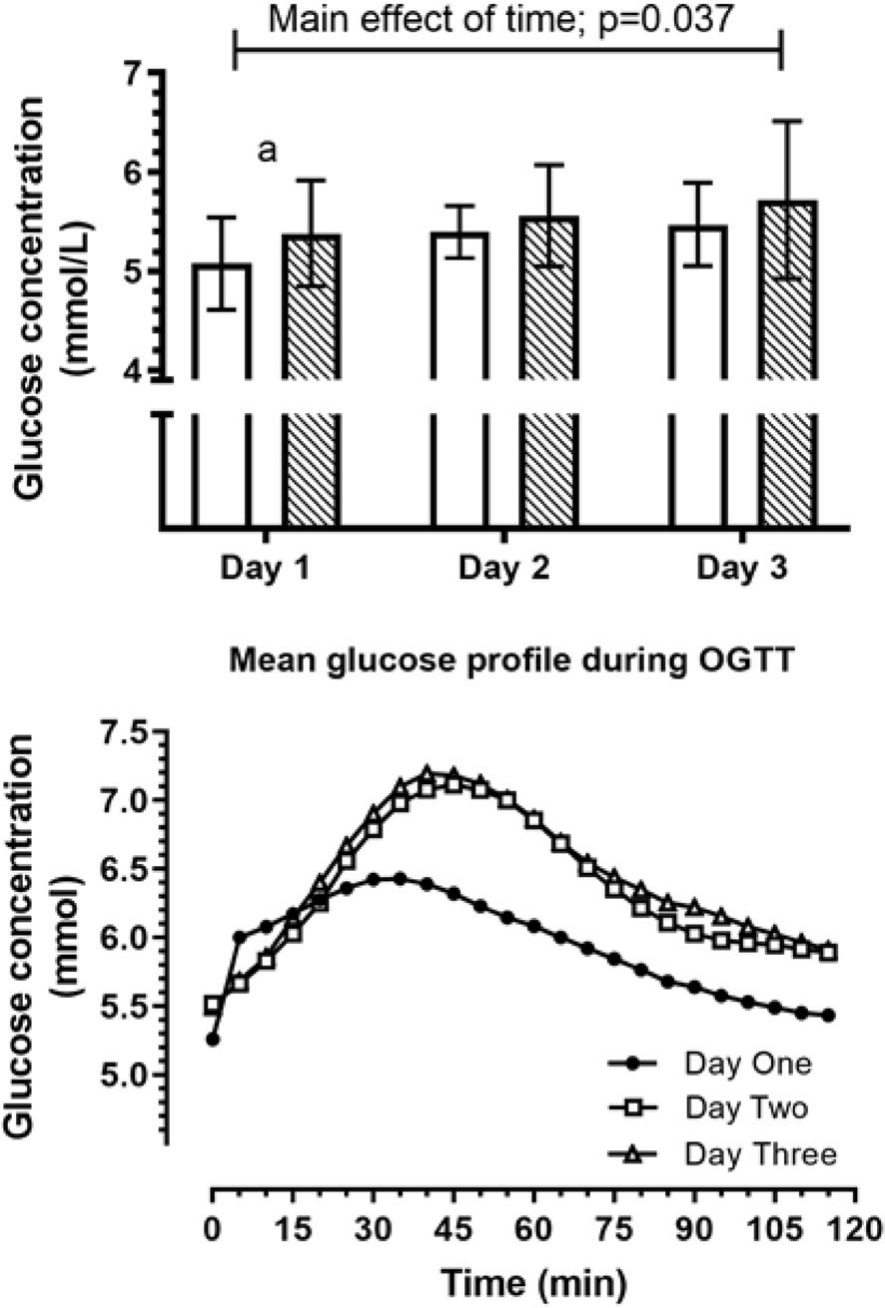
Glucose response measured using the continuous glucose meter (CGMS) during the OGTT on day 1 (control day), day 2 (60 min post-exercise) and day 3 (25 h post-exercise) are presented in the top panel. The mean glucose profile for 120 min post OGTT is presented for each day (Day 1, Day 2, Day 3), collapsed across conditions (i.e., CME and HIIE) in the bottom panel. a, significant difference between Day 1 and Day 3, *p* = 0.05

**Fig. 3:**
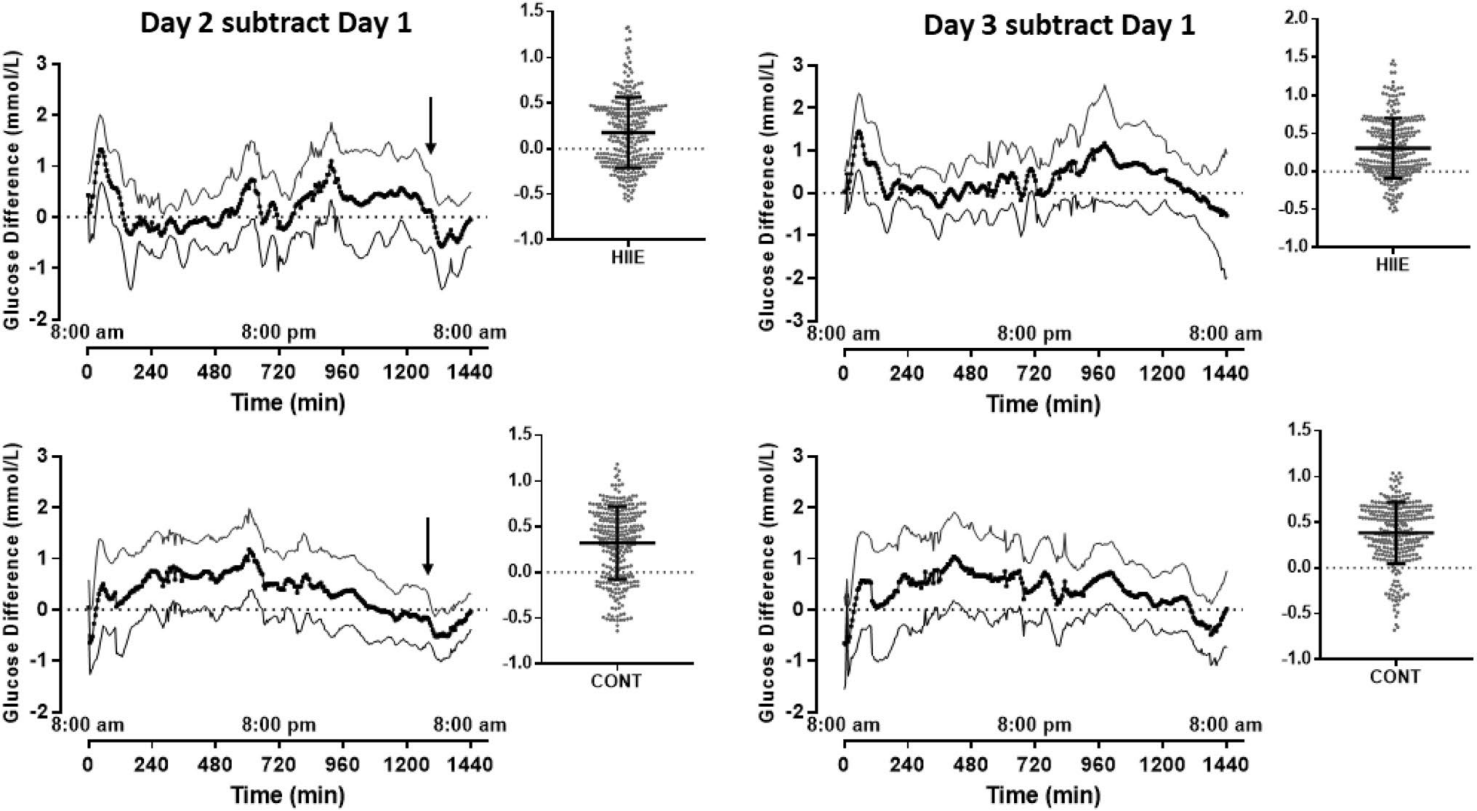
Mean differences (with 95% CI) in 24 h glucose concentrations between Day 2 and Day 1 in response to the HIIE (top panels) and continuous moderate intensity (bottom panels) exercise. Data are presented as top left: HIIE on Day-2 subtract Day-1; bottom left: CME on Day-2 subtract Day-1; top right: HIIE on Day-3 subtract Day-1; bottom right: CME on Day-3 subtract Day-1. Arrow indicates when exercise was completed. Subplot adjacent to each graph represents mean glucose difference concentration and standard deviation over each respective 24 h period along with the mean and 95% CI. Interpretation of this subplot is based on the 95% CI not crossing the zero-line (indicated by the dotted line). The subplot in the bottom right panel indicates the interstitial glucose concentration was higher on day 3 than on day 1

Following the evaluation of data from the CGMS, data from 3 participants were excluded due to significant (> 4 h) lapses in the measurements. There was a main effect of time (*p* = 0.037) on mean-daily glucose values, with the average glucose concentration on Day 3 being higher than Day 1 (Glucose 5.6 ± 0.2 vs Glucose 5.2 ± 0.1 mmol/L; *p* = 0.050), but there was no significant interaction effect (*p* = 0.755; Fig. 2, top). To assess the differences in the diurnal pattern of glucose between experimental days, comparisons between Day 2 (exercise) and Day 1 (baseline), and Day 3 (24 h recovery) and Day 1 were conducted by subtracting the Day 1 values, respectively (Fig. 3). There were only minor periods throughout the day, where significant differences between study days were observed as indicated by the upper- or lower-95% CI bands crossing zero (Fig. 3).

The glucose profile from the CGMS during the OGTT revealed a main effect of time (*p* < 0.001), but no condition by time interaction effects (*p* = 0.840; Fig. 2, bottom). The AUC-glucose revealed a significant effect of time (day; *p* = 0.027) but no interaction effect (*p* = 0.078). The AUC for glucose on Day 1 was significantly lower than day 2 (mean difference 0.28 mmol/L/min; value: *p* = 0.043) and day 3 (mean difference 0.46 mmol/L/min; *p* = 0.008).

## Discussion

This study sought to determine the responses in circulating APN, MMP-2, MMP-3 and OPN—key proteins involved in the remodelling of adipose tissue (Wu et al. 2017; Goncalves et al. 2015; Kahles et al. 2014; Sheikh et al. 2022; Unamuno et al. 2021; Akra et al. 2020)—following two different exercise formats in individuals with overweight or obesity. The main findings were (i) MMP-3 was acutely increased in response to exercise, while APN was not significantly different; (ii) MMP-2 did not demonstrate significant changes in response to exercise; (iii) OPN was acutely decreased following exercise, returning to baseline levels by 25 h. In addition, we sought to determine the interstitial glucose response over a 48 h period following the exercise session. The main findings were (i) exercise did not improve diurnal interstitial glucose profiles in this cohort; (ii) the glucose profile immediately following the OGTT supported the diurnal pattern, with the glucose-AUC performed 1 h after exercise (Day 2) and 25 h after exercise (Day 3) being significantly higher than the glucose-AUC on Day 1. In contrast to our hypothesis, the effect of HIIE did not appear to be greater than an energy-matched continuous exercise bout, on any measured marker in this study.

Elevated postprandial glucose is an independent risk factor for cardiovascular events in individuals with or without type 2 diabetes (Bonora and Muggeo 2001). Increased physical activity, however, has been shown to ameliorate the levels of hyperglycaemia (Mikus et al. 2012; Van Dijk et al. 2012). Despite some previous studies showing improvements in systemic glucose following acute exercise (Manders et al. 2010; Mikus et al. 2012; Van Dijk et al. 2012; Oberlin et al. 2014), neither a single bout of CME or HIIE in the present study was sufficient in reducing glucose concentrations under the current experimental conditions. A number of experimental differences may explain this discrepancy between studies. Specifically, the study of Oberlin et al., Manders et al. and Van Dijk et al., were performed in individuals with T2D (Van Dijk et al. 2012; Oberlin et al. 2014; Manders et al. 2010), while participants with overweight or obesity, but no glucose disturbances, were recruited in the current study. The study performed by Mikus et al. modified daily step count as the physical activity intervention (i.e., > 10,000 steps per day versus < 5000 steps per day for three successive days) rather than performing a single exercise session (Mikus et al. 2012). The timing of exercise and the energy intake may also have affected glucose responses (Teo et al. 2018, 2020), given the OGTT was conducted in a fasted state 1 h following exercise in the current study, rather than 2 h following exercise (Oberlin et al. 2014) or consuming breakfast (test meal) prior to exercise (Van Dijk et al. 2012).

There appears to be an important interplay between APN, MMPs and OPN during development of obesity and insulin resistance (Daniele et al. 2014). Among these proteins, the role of APN in the development of obesity and metabolic disease is the most well established, with the concentration of total APN decreasing with increasing adiposity and metabolic dysfunction (Nigro et al. 2014). Accordingly, the concentration of APN in the current study (~ 5 µg/ml) is at the lower end of the expected range (3–30 µg/ml) for human plasma. The acute effect of exercise on APN is equivocal with some reporting increases following acute moderate-to-high intensity (55–70% $\mathop {\text{V}}\limits^{.}$O_2max_) aerobic exercise (Kriketos et al. 2004; Numao et al. 2011), and others reporting no changes following moderate-intensity (60–65% $\mathop {\text{V}}\limits^{.}$O_2max_) aerobic exercise (Jamurtas et al. 2006; Magkos et al. 2010). In the current study, neither CME nor HIIE altered the concentration of circulating APN.

To our knowledge, this is the first study to have investigated the effect of exercise intensity on MMP-2 and MMP-3 in individuals with overweight or obesity. The transient increase in MMP-3 immediately post-exercise in the current study was similar to that observed in sedentary men (Urso et al. 2009), as well as endurance-trained and sedentary individuals (Schild et al. 2016). Although the response in MMP-3 to exercise in the study of Schild et al. was significantly more pronounced in endurance-trained individuals than sedentary individuals (Schild et al. 2016). In the current study, MMP-2 did not change in response to acute exercise, which is consistent with previous research reporting an acute increase in plasma MMP-2 concentration only in endurance-trained individuals, not in sedentary individuals (Schild et al. 2016).

The activation of MMPs is complex, requiring an initial cleavage from pro-MMP which can then be activated through oxidative stressors (Jaoude and Koh 2016) such as those expected during an acute, high-intensity exercise session. While MMPs play important roles in tissue remodelling, they may also inactivate cytokines and chemokines including interleukin-1β. It has been postulated that the acute increase in MMPs post-exercise, might function to diminish an excessive post-exercise pro-inflammatory response (Schild et al. 2016). While a main effect of condition was observed in the MMP-2 response, this was due to a higher baseline value where the interaction effect was not significant. Previous research using a cell culture system (Tong et al. 2011) found that APN may also induce the expression of MMP-3; however, in the present study, the transient increase in MMP-3 occurred without a significant change in APN concentration.

OPN appears to play an important initiating role in adipose-tissue expansion, inducing the expression of MMP-2 via pro-MMP-2, and inflammation (Daniele et al. 2014; Philip et al. 2001), as well as directly serving as a substrate for MMP-2 and MMP-3 (Lindsey et al. 2015). The acute decrease in OPN concentration immediately post-exercise coincided with the increase in MMP-3 concentration. This temporal association may explain the acute decrease in OPN immediately post-exercise, but further research is required.

Although previous findings of reduced OPN in mice was beneficial in reducing adipose tissue inflammation and improving glucose tolerance (Kiefer et al. 2010; Nomiyama et al. 2007), the reduction in OPN in the current study was only transient and appeared not to have an effect on glucose tolerance. Indeed, while the respective response of these markers to exercise are consistent with potentially longer-term health benefits; in the current study, these changes were not associated with changes in diurnal glucose profiles as evidenced by a lack of significance over time and the higher glucose responses 1 h and 25 h post-exercise (Figs. 2 and 3).

This study had a number of strengths including (i) duration- and workload-matched CME and HIIE conditions; (ii) recruiting a clinically relevant population, namely individuals who were inactive and overweight or obese- which has, to our knowledge not previously been conducted in a study of this kind; (iii) adopting a randomized cross-over design; (iv) each trial included a specific baseline compromising of 24 h continuous glucose monitoring which were used to compare 24 h differences following exercise for 2  days post-exercise. However, there were also some limitations associated with this study. Despite the measurement of MMP-2 and MMP-3 in this study, we did not measure activity of tissue inhibitors of MMPs which would have accounted for the regulatory activity of these MMPs which may have provided further insight into the acute effect of MMPs following exercise. Additionally, the measurement of MMPs and other proteins were limited to plasma or serum only. Simultaneous measurement of these proteins in the circulation, as well as multiple tissues including skeletal muscle and adipose tissue would provide further mechanistic insights regarding their exercise responses. Implementing a similar study in a population of individuals spanning the spectrum from normoglycemia to individuals with obesity and type 2 diabetes would be a strength for future studies and may allow assessment across clinical and pre-clinical stages.

Although the strict timing on completion of the OGTT allowed for a level of consistency over each trial day, providing all participants with standardized meal over the three days would have allowed for greater control of dietary intake. Similarly, controlling the levels of physical activity on each day (beyond the exercise bout), would be a strength for future research studies; although, based on our physical activity monitoring with a tri-axial activity monitor, energy expenditure, sedentary time and total vector counts appeared to be no different between exercise conditions or days. Finally, conducting multiple regression analysis to assess the effect of respective markers on glucose profile would provide more substantial and concrete answers; however, a greater number of participants would be required to run such an analysis. Due to the lack of significant change in the glucose profile following exercise, we were not able to determine the impact of the changes in these inflammatory markers on the glucose response.

## Conclusions

Important roles for MMP-2, MMP-3 and OPN in the remodeling of adipose tissue and development obesity-related diseases are now emerging (Wu et al. 2017; Goncalves et al. 2015; Kahles et al. 2014; Sheikh et al. 2022; Unamuno et al. 2021; Akra et al. 2020). The present study demonstrated that a single bout of moderate- or high-intensity intermittent exercise in individuals with overweight or obesity resulted in an acute and transient increase in MMP-3 and decrease in OPN. While these changes align with expected responses to acute exercise (MMP-3) and improvements in metabolic outcomes (OPN), continuous glucose concentrations measured for 48 h post-exercise under free-living conditions, were not different to the 24 h pre-exercise glucose concentrations under the current experimental conditions. Further research is warranted to assess the response of OPN and MMPs in response to exercise training, particularly in a cohort of individuals with T2D, along with a deeper characterization of the interaction of the MMPs and the various OPN isoforms.

## Data Availability

The datasets generated in the current study are available from the corresponding author on reasonable request.

## Abbreviations

APN: Adiponectin
AUC: Area under the curve
CGMS: Continuous glucose monitoring system
CME: Continuous moderate-intensity exercise
DXA: Dual-energy X-ray absorptiometry
HIIE: High-intensity intermittent exercise
MMPs: Matrix metalloproteinases
OPN: Osteopontin
T2D: Type 2 diabetes mellitus
$\mathop {\text{V}}\limits^{.}$O_2peak_: Peak pulmonary oxygen uptake
